# Maternal circadian disruption is associated with variation in placental DNA methylation

**DOI:** 10.1371/journal.pone.0215745

**Published:** 2019-04-26

**Authors:** Danielle A. Clarkson-Townsend, Todd M. Everson, Maya A. Deyssenroth, Amber A. Burt, Karen E. Hermetz, Ke Hao, Jia Chen, Carmen J. Marsit

**Affiliations:** 1 Department of Environmental Health, Rollins School of Public Health, Emory University, Atlanta, Georgia, United States of America; 2 Department of Environmental Medicine and Public Health, Icahn School of Medicine at Mount Sinai, New York, New York, United States of America; 3 Department of Genetics and Genomic Sciences, Icahn School of Medicine at Mount Sinai, New York, New York, United States of America; University of Lübeck, GERMANY

## Abstract

Circadian disruption is a common environmental and occupational exposure with public health consequences, but not much is known about whether circadian disruption affects *in utero* development. We investigated whether maternal circadian disruption, using night shift work as a proxy, is associated with variations in DNA methylation patterns of placental tissue in an epigenome-wide association study (EWAS) of night shift work. Here, we compared cytosine-guanosine dinucleotide (CpG) specific methylation genome-wide of placental tissue (measured with the Illumina 450K array) from participants (n = 237) in the Rhode Island Child Health Study (RICHS) who did (n = 53) and did not (n = 184) report working the night shift, using robust linear modeling and adjusting for maternal age, pre-pregnancy smoking, infant sex, maternal adversity, and putative cell mixture. Statistical analyses were adjusted for multiple comparisons and results presented with Bonferroni or Benjamini and Hochberg (BH) adjustment for false discovery rate. Night shift work was associated with differential methylation in placental tissue, including CpG sites in the genes *NAV1*, *SMPD1*, *TAPBP*, *CLEC16A*, *DIP2C*, *FAM172A*, and *PLEKHG6* (Bonferroni-adjusted p<0.05). CpG sites within *NAV1*, *MXRA8*, *GABRG1*, *PRDM16*, *WNT5A*, and *FOXG1* exhibited the most hypomethylation, while CpG sites within *TDO2*, *ADAMTSL3*, *DLX2*, and *SERPINA1* exhibited the most hypermethylation (BH q<0.10). Functional analysis indicated GO-terms associated with cell-cell adhesion and enriched GWAS results for psoriasis. Night shift work was associated with differential methylation of the placenta, which may have implications for fetal health and development. This is the first study to examine the epigenetic impacts of night shift exposure, as a proxy for circadian disruption, on placental methylation in humans, and, while results should be interpreted with caution, suggests circadian disruption may have epigenetic impacts.

## Introduction

Disruption of circadian rhythms is associated with negative health outcomes such as cancer, metabolic disorders, and neurological disorders in epidemiologic[1] and animal studies[2]; however, the impacts of circadian disruption during pregnancy on fetal development and child health have been largely overlooked. The core circadian clock consists of feedback loops of transcription factors (TF) that generate oscillating cycles of gene transcription and translation. These endogenously generated rhythms rely on cues, such as light, to synchronize patterns of physiological activity with the external environment. Light signals the suprachiasmatic nucleus (SCN) of the hypothalamus, the “master clock”, to set the body’s peripheral clocks[3].

There have been numerous studies to evaluate health outcomes associated with night shift work, an occupational proxy for circadian disruption, but it is unknown whether working the night shift before or during pregnancy poses health risks to the mother or child. This understudied exposure may have large public health consequences, as approximately 15% of American employees work outside of the traditional 9AM-5PM work schedule[4]. While some aspects of the circadian system may return to normal after a regular schedule of night shift work, studies suggest the majority of regular night shift workers (~97%) are not able to fully adapt their endogenous circadian rhythms to their work schedules[5].

Although there appear to be only small risks of negative reproductive health outcomes associated with shift work[6], not much is known about the impact of light or circadian rhythms in human pregnancy or on long-term fetal programming. The placenta, an organ responsible for mediating the maternal and fetal environment to regulate growth and development, may be affected by circadian disruption; yet, little attention has been paid to the impact of circadian disruption on placental function. Because the placenta is composed of fetal DNA, methylation of placental tissue may reflect fetal exposures and future health effects. Therefore, differences in placental methylation patterns between night shift workers and non-night shift workers may indicate altered fetal development and infant health in response to circadian disruption. In this study, we conducted an epigenome-wide association study (EWAS) to investigate whether night shift work is associated with differences in DNA methylation in the placental epigenome, which can impact long-term health outcomes in the offspring.

## Methods

### Study population—The Rhode Island Child Health Study (RICHS)

The Rhode Island Child Health Study (RICHS) is a hospital-based cohort study of mothers and infants in Rhode Island, described in detail elsewhere[7]. Briefly, from 2009 to 2014, women between the ages of 18–40 and their infants were enrolled at the Women and Infants Hospital of Rhode Island, oversampling for large and small for gestational age infants and matching each to an appropriate for gestational age control by maternal age (± 2 years), sex, and gestational age (± 3 days). RICHS enrolled only full-term (≥37 weeks), singleton deliveries without congenital or chromosomal abnormalities. All participants provided written informed consent under protocols approved by the Institutional Review Boards of Women and Infants Hospital and Emory University.

Demographic information was collected from a questionnaire administered by a trained interviewer and clinical outcome information was obtained from medical records. Information on night shift work was obtained from questionnaire by first asking, “Have you ever worked outside the home? (Yes/No)” and if “Yes”, participants were asked “If yes, please list all of the jobs you have had starting with your current job first. Please indicate whether you worked a swing shift or a night shift on any of these jobs”. To indicate shift jobs, the questionnaire included check boxes for “Yes” and “No” under a category for “Night Shift”. For this analysis, only the most recently reported job history was used and only those who reported “Yes” for night shift work were considered night shift workers; people who reported working a swing shift but not a night shift were not included as night shift workers.

To adjust for socioeconomic factors while avoiding multicollinearity, we used an adversity score index to adjust for household income, maternal education, marital status and partner support. The cumulative risk score ranged from 0 to 4, with 0 representing the lowest level of adversity and 4 representing the highest level of adversity. A higher risk score was given to women whose median household income (adjusting for the number of people in the household) fell below the federal poverty line for the year the infant was born (+1), to women whose household was larger than 6 (+1), to women who were single and did not receive support from a partner (+1) and to women whose highest level of education was high school or less (+1)[8].

### Placental sample collection and measurement of DNA methylation

Genome-wide DNA methylation (measured with the Illumina 450k arrays) was obtained on 334 placentae parenchyma samples in RICHS as previously described[9], and of these, data from 237 samples were included in this analysis. The QA/QC process has been described elsewhere[10], including functional normalization, BMIQ, and ‘ComBAT’ to adjust for technical variations and batch effects in R[9, 11]. Briefly, we used the ‘minfi’ package in R to convert the raw methylation files to β values, a ratio of methylation ranging from 0 to 1, for analysis. Probes associated with the X or Y chromosomes, single nucleotide polymorphism (SNP)-associated (within 10bp of the target cytosine-guanosine dinucleotide (CpG) site and with minor allele frequency >1%), identified as cross-reactive or polymorphic by Chen et al[12], or with poor detection p-values were excluded, yielding 334,692 probes for analysis in this study[9]. DNA methylation array data for RICHS can be found in the NCBI Gene Expression Omnibus (GEO) database with the number GSE75248. Women with missing information on pre-pregnancy smoking status (“No”/”Yes”), defined as smoking 3 months prior to pregnancy, or adversity score were not included in the analysis. Women who did not provide an answer for the nightshift variable (n = 16) were recoded to “No”. This study included the 237 mother-infant pairs within RICHS for which DNA methylation data and the necessary demographic information were available.

### Placental RNA sequencing

Gene expression was measured using the Illumina HiSeq 2500 system in 199 placental samples from RICHS; methods have been previously described [13]. After standard QA/QC procedures, final data were normalized to log2 counts per million (logCPM) values. Raw data is available in the NCBI sequence read archive (SRP095910).

### Statistical analyses

Because a reference panel for placental cell types does not yet exist, we used a validated reference-free method, the ‘RefFreeEWAS’ package in R, to adjust for heterogeneity in cell-type composition[14, 15]. We implemented the RefFree estimation via the same process described in detail in our lab’s prior work[16], and identified 8 components to represent the putative cell mixture in our placental samples. We also examined the outlier screening plots of the cell mixture array for extreme outliers. We then conducted an EWAS using robust linear modeling by regressing CpG methylation β-values on night shift work (“No”/”Yes”), adjusting for putative cell mixture, maternal age (years), pre-pregnancy smoking status (“No”/”Yes”) adversity score (0–4)[17], and sex of the infant (“Female”/”Male”). To adjust for multiple comparisons, we used the Bonferroni method and the Benjamini and Hochberg (BH) false discovery rate (FDR) methods. To evaluate the extent of *in utero* night shift exposure, we compared job and delivery date data. A sensitivity analysis using data from women who provided night shift job information (n = 221) without recoding missing to “No” was performed. We also conducted a sensitivity analysis to evaluate gestational diabetes mellitus (GDM) and CpG methylation outcomes, as numerous studies have found an association between night shift work and the development of obesity and metabolic diseases[18, 19], as well as GDM and altered methylation[20]. While DNA methylation is not expected to change on a day-to-night basis[21], a few recent studies of brain tissue have found diurnal differences in DNA methylation[22, 23]. To assess possible confounding by time of placenta sample collection, we categorized time of sample collection into 3-hour bins (7AM-9AM, 10AM-12PM, 1PM-3PM, and 4PM-5PM) and performed a Fisher’s exact test (one of the cells had <5 participants) to compare night shift and non-night shift workers. We also modelled sample collection time as a continuous outcome and night shift work as a categorical exposure (No/Yes).

Additionally, we investigated differentially methylated regions (DMRs) using the ‘Bumphunter’ package in R[24]. We modeled the β-values between non-night shift workers and night shift workers, controlling for the same variables as the individual CpG by CpG site genome-wide analysis. CpG sites within 500 base pairs were clustered together and β-values were modeled against a null distribution generated via bootstrapping; sites with differential methylation of 2% or more were considered to be possible DMRs.

To examine the functional implications of night shift work-associated DNA methylation (BH q<0.05), we also conducted an expression quantitative trait (eQTM) analysis using ‘MEAL’[25] in R to investigate whether methylation was associated with gene expression in the RICHS samples on which both DNA methylation and expression data were available (n = 199). Using robust linear modeling, we regressed the expression levels of genes within a 100kb window of the CpG site on methylation β-values (p<0.05).

### Bioinformatic analyses

To better understand the biological significance of the EWAS results, we performed an enrichment analysis of the top 298 CpG sites (BH q<0.10) with GO-terms and KEGG pathways in R using the ‘missMethyl’ package[26]. We also evaluated whether the genes (n = 45) from overlapping CpG sites with BH q<0.05 were listed as rhythmic within the available CircaDB mouse databases[27]. We searched within CIRCA mouse experimental datasets using the JTK filter with a q-value probability cut-off of 0.05 and a JTK phase range of 0–40[28]. To investigate whether the CpGs from our EWAS results were within genomic regions that have been linked to traits from previous GWAS findings, windows of the top 298 CpG sites (BH q<0.10) and flanking 5kb regions of DNA were compared for overlap with SNP results (p<1x10^-8^) in the GWAS catalog of the National Human Genome Research Institute and the European Bioinformatics Institute (NHGRI-EBI)[29] using the TraseR package[30] in R. As background, we only included SNPs that were within 5kb of CpGs that were included in the EWAS study. If more than one top CpG site fell within the same 10kb window, they were clustered together and considered the same region. Only GWAS trait-associated SNP windows that overlapped with 2 or more of the top CpG windows and were statistically significant after Fisher’s exact test (BH q <0.05) were considered enriched.

## Results

### Demographic and medical information

Demographic information for the women (n = 237) and covariates included in the final model for the epigenome-wide analysis are provided in Table 1. A sensitivity analysis comparing results from women who provided night shift job information (n = 221) without recoding missing to “No” did not indicate any large differences in demographic features. Overall, women who reported working the night shift were more likely to be younger, smokers pre-pregnancy, cases of GDM, single and never married, lower household income, and higher adversity (p<0.05). While not statistically significant, women who worked the night shift trended towards a higher BMI and an evening chronotype. Of those included in the analysis, one participant reported taking melatonin and she was not a night shift worker. Additionally, 37 out of the 53 (70%) night shift workers reported working the night shift during pregnancy; time between working the night shift and the birth of the infant ranged from within a week to approximately 4.5 years, with a median value of 10 weeks.

**Table 1:** Demographic characteristics of participants included in the analysis (n = 237) by night shift work status.

	N	Non-night shift (n = 184)	Night shift (n = 53)	Statistical significance
Maternal age*, mean ± SD	237	30.7+/- 5.4	28.8+/- 5.1	p<0.05
Pre-pregnancy smoking*, % (n)	237	12% (22)	25% (13)	p<0.05
Sex of the infant (male/female), % (n)	237	48% / 52% (88/96)	53% / 47% (28/25)	p = 0.6
Gestational diabetes*, % (n)	234	9% (16)	21% (11)	p<0.05
Marital status*, % (n)	237			p<0.05
Single, never married		24% (44)	43% (23)	
Separated or divorced		3% (6)	4% (2)	
Married		73% (134)	53% (28)	
Household income*, % (n)	229			p<0.01
<$9–14,999		14% (25)	24% (12)	
$15–29,999		9% (17)	20% (10)	
$30–49,999		10% (18)	18% (9)	
$50–99,999		39% (69)	30% (15)	
.>$100,000		28% (50)	8% (4)	
Adversity score*, % (n)	237			p<0.05
0		78% (143)	58% (31)	
1		12% (22)	30% (16)	
2		9% (16)	9% (5)	
3		1% (2)	2% (1)	
4		1% (1)	0% (0)	
Maternal education*, % (n)	237			p<0.01
<11^th^ grade		5% (10)	4% (2)	
High school		15% (28)	26% (14)	
Junior college or equivalent		22% (40)	40% (21)	
College		36% (67)	26% (14)	
Any post-graduate		21% (39)	4% (2)	

*Signifies p-value <0.05 (using either χ^2^ test, Fisher’s exact test or 2-sided t-test) between non-night shift and night shift workers.

### Epigenome-wide methylation associations

DNA methylation at 298 CpG sites was found to be significantly different in night shift workers after FDR correction at the BH q<0.10, 57 CpG sites significant at the BH q<0.05 (Table 2), and 10 CpG sites at the Bonferroni-corrected p<0.05 (Table 2). CpG sites for the *NAV1*, *SMPD1*, *TAPBP*, *CLEC16A*, *DIP2C*, *FAM172A*, and *PLEKHG6* genes had genome-wide significance after Bonferroni correction (p<0.05). The *ADAMTS10*, *CLEC16A*, *CTBP1*, *EGFL8*, *GNAS*, *HDAC4*, *HEATR2*, *KCNA4*, *KDELC2*, *MFHAS1*, *MXRA8*, *NAV1*, *PLXND1*, *UBR5*, *WNT5A*, and *ZBTB22* genes had multiple CpG sites represented in the results.

**Table 2:** List of differentially methylated CpG sites in night shift workers compared to non-night shift workers after epigenome-wide analysis (BH q<0.05).

UCSC Gene Name	Chromosome	Position	Probe ID	UCSC Gene Group	Enhancer	β1	SE	P-value	BH q-value	Bonferroni
*NAV1*	chr1	201708718	cg14168733	TSS1500	NA	-0.04	0.007	2.53E-08	0.003	0.008
*NAV1*	chr1	201709135	cg14377596	1stExon	TRUE	-0.04	0.007	2.98E-08	0.003	0.01
*SMPD1*	chr11	6412852	cg14814323	Body	NA	-0.016	0.003	2.97E-08	0.003	0.01
*NAV1*	chr1	201709390	cg01411786	Body	TRUE	-0.032	0.006	9.91E-08	0.004	0.033
*TAPBP*	chr6	33273011	cg03190911	Body	NA	-0.014	0.003	9.94E-08	0.004	0.033
	chr6	27390647	cg06667732		NA	-0.023	0.004	9.35E-08	0.004	0.031
*CLEC16A*	chr16	11073063	cg08082763	Body	TRUE	-0.023	0.004	7.21E-08	0.004	0.024
*DIP2C*	chr10	560669	cg21373996	Body	NA	-0.019	0.004	1.06E-07	0.004	0.035
*FAM172A*	chr5	93076910	cg25342875	Body	NA	-0.024	0.004	9.46E-08	0.004	0.032
*PLEKHG6*	chr12	6436676	cg14858786	Body	NA	-0.026	0.005	1.42E-07	0.005	0.047
*KRT15*	chr17	39675154	cg11983245	5'UTR	NA	-0.024	0.005	1.84E-07	0.005	0.062
*NAV1*	chr1	201709675	cg18539461	Body	TRUE	-0.036	0.007	1.71E-07	0.005	0.057
*RHOT2*	chr16	717556	cg04365973	TSS1500	NA	-0.019	0.004	2.58E-07	0.007	0.086
*NAV1*	chr1	201708888	cg13877974	TSS200	NA	-0.043	0.009	4.11E-07	0.01	0.137
*ERI3*	chr1	44716226	cg24373865	Body	NA	-0.024	0.005	5.66E-07	0.013	0.189
*PTPN6*	chr12	7060187	cg23147227	TSS1500	NA	-0.02	0.004	8.98E-07	0.019	0.301
*EGFL8*	chr6	32135718	cg08759957	Body	NA	-0.021	0.004	1.22E-06	0.023	0.407
*ZBTB22*	chr6	33284168	cg14771240	Body	NA	-0.02	0.004	1.18E-06	0.023	0.396
	chr10	22725309	cg01422243		NA	-0.019	0.004	1.51E-06	0.027	0.504
*UBR5*	chr8	103344822	cg02530407	Body	TRUE	-0.019	0.004	1.68E-06	0.027	0.561
*HDAC4*	chr2	240213173	cg23601374	Body	TRUE	-0.017	0.004	1.64E-06	0.027	0.549
	chr7	25702848	cg03700230		NA	0.048	0.01	1.93E-06	0.029	0.645
*CYB5R2*	chr11	7694163	cg05919312	5'UTR	NA	-0.018	0.004	2.03E-06	0.03	0.679
*RPS6KA4*	chr11	64139406	cg07425109	3'UTR	NA	-0.016	0.003	2.15E-06	0.03	0.719
*MSI2*	chr17	55742491	cg07618409	Body	TRUE	-0.02	0.004	2.26E-06	0.03	0.755
*CDYL2*	chr16	80716710	cg16713168	Body	TRUE	-0.021	0.004	2.33E-06	0.03	0.78
*FAM118A*	chr22	45705265	cg06575572	5'UTR	NA	-0.02	0.004	2.50E-06	0.03	0.835
*LRRC2*	chr3	46618325	cg07225641	5'UTR	NA	-0.027	0.006	2.61E-06	0.03	0.875
*CLDN9*	chr16	3063894	cg10492999	1stExon	NA	-0.026	0.006	2.70E-06	0.03	0.905
*LOC645323*	chr5	87955859	cg13982098	Body	NA	-0.028	0.006	2.65E-06	0.03	0.886
*C2orf54*	chr2	241827789	cg21333033	Body	NA	-0.019	0.004	2.93E-06	0.032	0.981
*SLC41A1*	chr1	205780033	cg00762738	5'UTR	NA	-0.017	0.004	3.08E-06	0.032	1
*MXRA8*	chr1	1290712	cg00040588	Body	NA	-0.051	0.011	3.49E-06	0.032	1
*EGFL8*	chr6	32135715	cg12305588	Body	NA	-0.019	0.004	3.35E-06	0.032	1
*BAIAP2*	chr17	79022879	cg12472449	Body	NA	-0.016	0.004	3.43E-06	0.032	1
*FBXW7*	chr4	153437193	cg13536107	5'UTR	TRUE	-0.022	0.005	3.35E-06	0.032	1
*BAT2*	chr6	31599646	cg25371129	Body	NA	-0.005	0.001	3.61E-06	0.033	1
*MIRLET7A3*	chr22	46508563	cg04063235	TSS200	NA	-0.019	0.004	3.71E-06	0.033	1
*HDLBP*	chr2	242174625	cg11221200	Body	NA	-0.014	0.003	3.90E-06	0.033	1
	chr11	22454301	cg23181580		TRUE	-0.031	0.007	4.22E-06	0.035	1
*BATF3*	chr1	212874153	cg00168835	TSS1500	NA	0.005	0.001	4.42E-06	0.036	1
	chr22	50221949	cg08174792		NA	-0.034	0.007	4.91E-06	0.039	1
*MFHAS1*	chr8	8749074	cg01022370	1stExon	TRUE	-0.023	0.005	5.20E-06	0.04	1
*ZNF284*	chr19	44575547	cg05333740	TSS1500	NA	-0.023	0.005	5.83E-06	0.042	1
*DPEP2*	chr16	68027297	cg06866814	5'UTR	NA	0.002	0	5.50E-06	0.042	1
*GALNTL4*	chr11	11438208	cg16337763	Body	TRUE	-0.022	0.005	5.67E-06	0.042	1
*AZI1*	chr17	79184968	cg20296990	Body	NA	-0.02	0.004	5.84E-06	0.042	1
*GALNTL1*	chr14	69725831	cg00080706	TSS1500	NA	-0.019	0.004	5.99E-06	0.042	1
*MFHAS1*	chr8	8749278	cg01784220	1stExon	TRUE	-0.022	0.005	6.21E-06	0.042	1
*C11orf2*	chr11	64863151	cg13626866	TSS1500	NA	-0.026	0.006	6.37E-06	0.043	1
*BANF1*	chr11	65770987	cg17985854	Body	NA	-0.023	0.005	6.49E-06	0.043	1
*IQGAP2*	chr5	75784957	cg23289545	Body	TRUE	-0.019	0.004	6.62E-06	0.043	1
	chr17	43222106	cg00625783		TRUE	-0.025	0.006	7.26E-06	0.045	1
*TUBGCP2*	chr10	135120640	cg04070692	5'UTR	NA	-0.019	0.004	7.21E-06	0.045	1
*BAT1*	chr6	31502388	cg10895184	Body	NA	-0.018	0.004	7.53E-06	0.045	1
*HAPLN1*	chr5	83016779	cg18024167	1stExon	NA	-0.023	0.005	7.44E-06	0.045	1
*PKHD1L1*	chr8	110374866	cg19906741	1stExon	TRUE	0.018	0.004	7.77E-06	0.046	1

**Fig 1:**
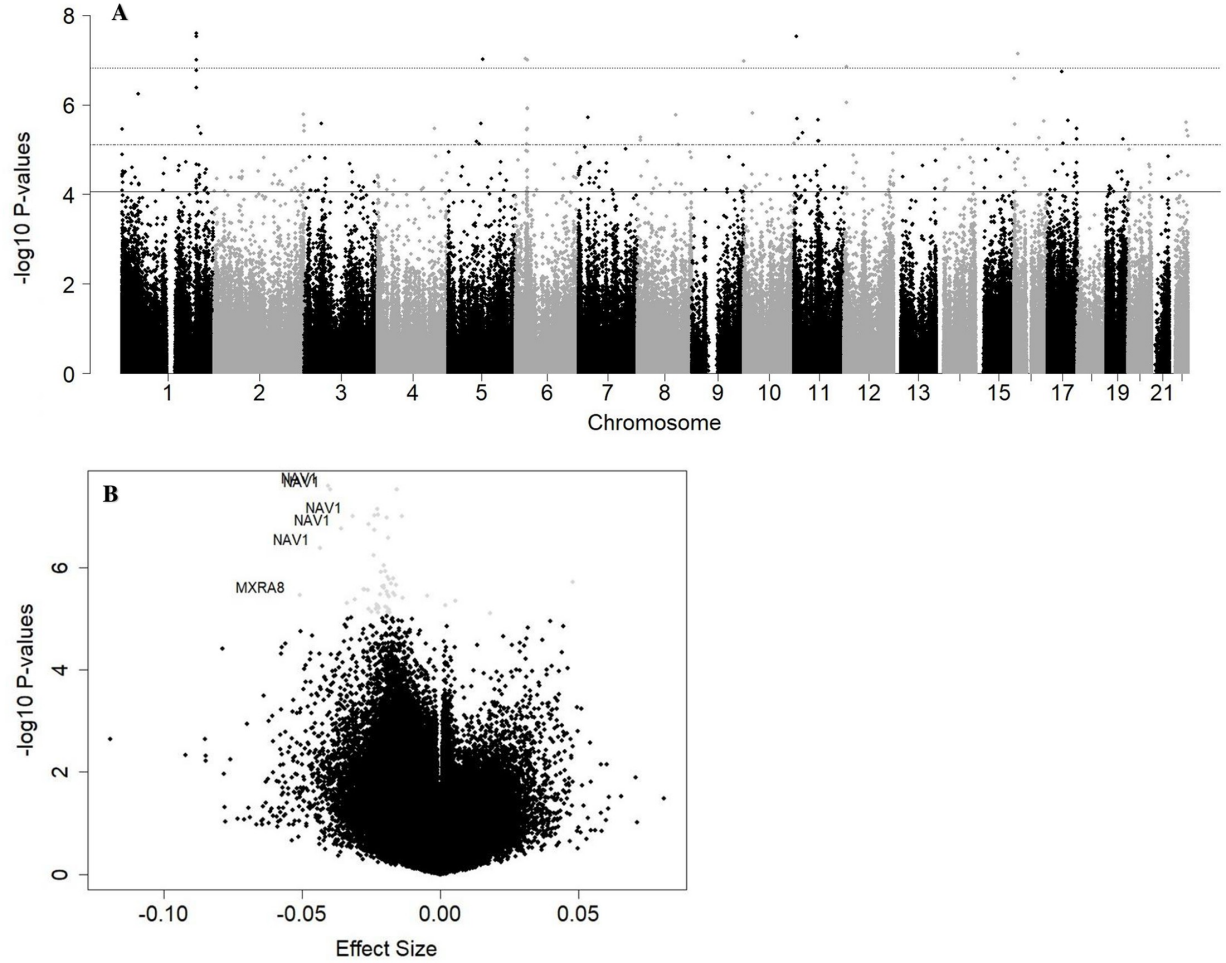
Results of placental DNA methylation and night shift work EWAS. **A,** Manhattan plot of CpG results, adjusted for maternal age, pre-pregnancy smoking, adversity score, sex of the infant, and estimated cell mixture. The dashed upper boundary line denotes p-value of 1.49x10-7 as the significance threshold after Bonferroni adjustment (p<0.05), the dashed middle boundary line denotes the p-value of 7.7x10-6 as the approximate significance threshold of BH q<0.05, and the solid boundary line at denotes the p-value of 8.8x10-5 as the approximate significance threshold of BH q<0.10. **B,** Volcano plot of results, adjusted for maternal age, pre-pregnancy smoking, adversity score, sex of the infant, and estimated cell mixture. Gray dots signify CpG sites with BH q<0.05 and CpG sites with both absolute beta coefficients of 0.03 or greater and BH q<0.05 are labelled with UCSC gene names.

The Manhattan plot of the results indicated a number of differentially methylated sites that distributed across the genome, with some occurring in the same regions (Fig 1A). There was also an overall trend towards hypomethylation (Fig 1B). CpG sites for *NAV1*, *MXRA8*, *GABRG1*, *PRDM16*, *WNT5A*, and *FOXG1* were among the 10 sites with the most hypomethylation, while CpG sites for *TDO2*, *ADAMTSL3*, *DLX2*, and *SERPINA1* were among the 10 sites with the most hypermethylation To more rigorously examine the co-located CpG sites associated with night shift work, we employed a ‘Bumphunter’ analysis and identified 6584 ‘bumps’, with areas of the *NAV1*, *PURA*, *C6orf47*, and *GNAS* genes as DMRs (BH q<0.10)(Table 3). Of these, CpGs for the *NAV1* and *GNAS* genes were also differentially methylated in the CpG by CpG analysis (S1 Table).

**Table 3:** ‘Bumphunter’ results of significant DMRs (BH q<0.10).

Gene	Chromosome	Start	End	β1	Area	L	clusterL	P-value	FWER	P-value Area	FWER Area	BH q-value	Bonferroni
*NAV1*	chr1	201708500	201709675	-0.038	0.452	12	12	3.29E-05	0.166	1.74E-04	0.606	0.054	0.217
*PURA*	chr5	139493486	139494006	-0.054	0.544	10	10	2.54E-05	0.131	6.80E-05	0.31	0.054	0.167
*C6orf47*	chr6	31627678	31627678	-0.112	0.112	1	38	3.24E-05	0.163	1.17E-02	1	0.054	0.213
*GNAS*	chr20	57463325	57463725	-0.034	0.482	14	30	9.35E-06	0.05	1.31E-04	0.512	0.054	0.062

A sensitivity analysis with GDM included as an additional covariate shared many of the top CpG sites with the primary results, suggesting GDM is not a confounder of these associations. An additional analysis evaluating GDM as the primary exposure shared no top genes with the EWAS results. In comparing time of placenta sample collection, there was no significant difference between the two groups when compared categorically (p = 0.547) or continuously (p = 0.945), with a mean collection time around 11AM. In another sensitivity analysis comparing the beta coefficients from models that utilized *in utero* night shift work exposure as the independent variable (n = 37) to the beta coefficients from models that included all night shift workers (n = 53), the differences were small; only 1 CpG site, cg24373865, had an absolute difference in beta coefficients greater than 0.01, at 0.011. We also re-examined our results removing those with missing data on night shift work and the findings were substantially similar.

### Functional analyses

Comparing the 298 significant CpG sites (BH q<0.10) to the remaining 334,394 CpG sites, there was a higher frequency of top CpG sites within enhancer regions (χ^2^ = 13.48, df = 1, p-value = 0.0002). Because transcription factors (TFs) can bind to enhancer regions to alter gene expression, we assessed whether CpG methylation was associated with expression levels in nearby genes. The eQTM analysis found the expression of 18 genes to be associated with 14 CpG sites (p<0.05). Of these, the expression levels of *ACBD4* were associated with methylation in cg00625783 (β1 = 2.515, p-value = 1.94E-05) and the expression levels of *KRT15* were associated with methylation in cg11983245 (β1 = 7.895, p-value = 8.04E-05)(S2 Table). For both of these genes, increasing methylation of the CpG sites was associated with increased gene expression. The cg00625783 CpG is not annotated to a gene, but is located within an enhancer region, and cg11983245 is annotated to the 5’ untranslated region (5’UTR) and 1^st^ exon of the *KRT15* gene. Methylation of cg11983245 was also associated (p<0.05) with increased *KRT19* (β1 = 4.404, p-value = 3.87E-03) and *LINC00974* (β1 = 6.011, p-value = 3.40E-02) expression levels.

We analyzed the top 298 CpG sites (BH q<0.10) for enrichment of KEGG pathways and GO-terms. The GO-terms “cell-cell adhesion”, “cell-cell adhesion via plasma-membrane adhesion molecules”, and “hemophilic cell adhesion via plasma membrane adhesion molecules” were found to be significant after FDR correction (BH<0.05)(S3 Table). The top KEGG pathway results were “valine, leucine and isoleucine biosynthesis”, “mucin type O-glycan biosynthesis” and “melanogenesis”, but they were not significant after correcting for FDR (S3 Table). Surprisingly, *PER1* was the only core circadian gene represented among the 298 CpG sites. However, we evaluated whether the 45 genes of the top 57 CpG sites exhibited circadian rhythmicity within the CircaDB mouse expression database[27] and found 27 out of the 45 genes (60%) displayed rhythmic expression[28](S4 Table). Of these genes, *BAIAP2*, *GALNTL1*, *HDLBP*, *NAV1*, and *TAPBP* displayed rhythmicity in mouse SCN tissue. We then tested for trait-associated SNP enrichment within 10kb regions surrounding the top 298 CpG sites (BH q<0.05) among GWAS SNPs (p<1x10^-8^) in NHGRI-EBI GWAS catalog[29] using a Fisher’s exact test in TraseR[30]. These regions were significantly enriched (FDR < 5%) for the following traits: Psoriasis, Systemic Lupus Erythematosus, Type 1 Diabetes Mellitus, and Multiple Sclerosis (S5 Table).

## Discussion

We identified a number of CpG sites exhibiting differential methylation associated with night shift work in newborn placental tissue. While the average absolute differences for the 298 CpG site corresponded to a roughly 1.7% change in methylation, even a small change in methylation may have physiologically-relevant effects, and these magnitudes of association are comparable to others reported for exposures including toxic trace elements and maternal smoking during pregnancy[31]. The overall trend of hypomethylation with night shift work may be due to increased TF binding to DNA, leading to chromatin changes establishing the hypomethylated state[32]. Because one of the core components of the circadian clock, CLOCK, acts as a histone acetyltransferase[33], it is also possible that circadian disruption impacts the epigenetic activity of CLOCK, affecting chromatin state and accessibility. However, there is still much to discover about circadian interactions with methylation and developmental processes.

Light at night and night shift work exposure can cause altered hormonal signaling and endocrine disruption; because hormone receptors can act as TFs, it is possible that circadian disruption causes increased hormonal signaling and increased TF binding. Animal studies of *in utero* circadian disruption suggest that circadian disruption may negatively affect the health and development of offspring[34]. For example, chronic changes in the photoperiod of pregnant rats caused increased leptin levels, insulin secretion, fat deposition, and decreased glucose tolerance of offspring in adulthood[35]. Additionally, mice exposed to a 22-hour light-dark cycle, instead of the normal 24-hour cycle, had altered methylation patterns in the SCN and altered circadian behavior; differential methylation was also found for genes related to axonal migration, synaptogenesis, and neuroendocrine hormones[36].

We identified a DMR and multiple individual CpG sites within and nearby to *NAV1* that were consistently represented among the top results. In general, the functions of NAV1, particularly in the placenta, are not well characterized. *NAV1* is homologous to the *unc-53* gene in *C*.*elegans*, which plays a role in axonal migration[37]. The mouse homolog also appears to play a role in neuronal migration; NAV1 is enriched in growth cones and associates with microtubule plus ends[38], and the deficit of *Nav1* causes loss of direction in leading processes[39]. Research has also found increased embryonic lethality, decreased birthweight, and infertility in female offspring for Nav1^-/-^ mice[40], suggesting an important role for *Nav1* in fetal development and health. Our mouse tissue query of the CircaDB database revealed that *Nav1* specifically displayed circadian rhythmicity in mouse SCN tissue (S4 Table). This suggests NAV1 may play a role in the mammalian SCN. A DMR was also identified in *GNAS*, which is imprinted in the paraventricular nucleus of the hypothalamus and encodes the G_s_α G-protein, which regulates cAMP generation and metabolism. *Gnas* is implicated in REM and NREM sleep and the browning of white adipose tissue for thermogenesis[41]. Additionally, in a microarray analysis of retina samples from an *rd/rd* mouse model, *Gnas* was implicated in melanopsin signaling[42]. Therefore, GNAS may be important in integrating light and metabolic cues.

The top 298 CpG results (BH q <.05) were enriched for traits related to psoriasis, lupus, type 1 diabetes, and multiple sclerosis, all of which involve the immune system and/or inflammation. Interestingly, a large study of night shift workers in the Nurse’s Health Study found an increased risk of psoriasis among night shift workers[43]. Another study that analyzed two separate cohorts also found an association between engaging in shift work before 20 years old and multiple sclerosis[44]. The skin has circadian rhythms that may affect the development of psoriasis[45], during which abnormal activity of keratinocytes and T cells can cause lesions. Because adhesion molecules may play an important role in this process, this GWAS trait may explain the KEGG-pathway and GO-term enrichment analysis, among which “cell-cell adhesion” and “melanogenesis” were some of the top results.

A possible limitation of this analysis is the moderate sample size of night shift workers (n = 53). Because placenta samples were only collected during daytime hospital hours (7AM-5PM), we are also limited in our ability to fully evaluate diurnal differences in DNA methylation. Additionally, the adjustment for cell-type heterogeneity is an estimation, so there is a possibility of residual confounding by cell type. On the other hand, the results may be a conservative estimate of the true association, as this analysis occurred in full-term pregnancies and approximately 30% of the women included as night shift workers did not have *in utero* exposure. While a sensitivity analysis of *in utero* night shift work exposure did not find large differences in the magnitudes of association, exposure to circadian disruption at different windows of development could have different magnitudes of effect. Prior research has found that shift workers continue to have chronic health effects even after they switch to a day shift schedule. For example, researchers found that a history of shift work was associated with a decrease in cognitive ability that took 5 years or more after cessation of shift work to recover[46]; this suggests recovery from regular shift work may take an extended period of time and a history of shift work may have a prolonged influence on health.

This is the first study to examine the epigenetic impacts of night shift exposure on placental methylation in humans, and results should be interpreted with caution. Methylation of placental tissue, an indicator of the *in utero* epigenetic landscape, reflects functional activities of the placenta, which can impact various aspects of fetal development, including neurodevelopment. The findings that the methylation of *NAV1* differed by night shift work exposure and that *Nav1* is rhythmically expressed in mouse SCN suggests NAV1 may play a role in the human circadian system. Because the circadian system coordinates an array of physiological systems, alterations to circadian system development could affect immune response, sleep patterns, behavior, metabolism, and future health status. We have found night shift work to be associated with variation in methylation of placental tissue, which has implications for fetal development and future health. However, these findings may also be relevant for people who experience circadian disruption due to common exposures such as light at night[47].

In conclusion, night shift work is associated with differential methylation patterns in placental tissue. NAV1 may be an important component in the development of the human circadian system. Night shift work is a complex exposure encompassing altered hormonal signaling, eating and activity patterns, light exposure, and sleep patterns. Therefore, it is difficult to tease apart which aspects of night shift work contribute to which result. However, night shift work is a prevalent exposure in the workforce and, more generally, circadian disruption is a common facet of modern life. Circadian disruption may contribute to immune-mediated and inflammatory disease, but it is still unclear how this exposure may affect fetal development and infant health. These findings warrant further investigation to evaluate the effects of *in utero* circadian disruption and possible impacts on fetal and child health, as well as the role of the circadian system in the function of the placenta.

## Supporting information

S1 Table List of the 298 CpG sites that were differentially methylated in placenta tissue from night shift workers compared to non-night shift workers (BH q<0.10). (XLSX)

S2 Table Table of results from expression quantitative trait methylation (eQTM) analysis of genes within 100kb of CpG sites with BH q<0.05. The data used in this analysis came from RICHS samples that had both methylation and RNAseq data available (n = 199).
(XLSX)

S3 Table Table of top results from the GO-term and KEGG pathway analyses of top 298 CpG sites (BH q<0.10). N refers to the overall number of genes annotated to the term or pathway and DE refers to the number of genes from the top CpG sites within that term or pathway.
(XLSX)

S4 Table Table of results from CircaDB analysis of EWAS gene results (n = 45 genes, q<0.05). Gene list was entered and analyzed using a JTK q-value filter with a probability cut-off of 0.05 and JTK phase range of 0–40 of all available CIRCA mouse databases.
(XLSX)

S5 Table Trait-associated SNP enrichment within 10kb regions surrounding the top 298 CpG sites (BH q<0.05) among GWAS SNPs in NHGRI-EBI GWAS catalog that yielded trait-associations at p<1x10-8. (XLSX)
